# Small PLGA nanocapsules Co-encapsulating copper sulfide nanodots and fluorocarbon compound for photoacoustic imaging-guided HIFU synergistic therapy†

**DOI:** 10.1039/c7ra12074e

**Published:** 2018-01-24

**Authors:** Minghua Yao, Ming Ma, Huixiong Xu, Xiaoxia Pan, Guang Xu, Rong Wu

**Affiliations:** Department of Ultrasound in Medicine, Shanghai Tenth People's Hospital, Tongji University School of Medicine 301 Yanchangzhong Road Shanghai 200072 People's Republic of China wurong7111@163.com; State Key Laboratory of High Performance Ceramics and Superfine Microstructures, Shanghai Institute of Ceramics, Chinese Academy of Sciences 1295 Dingxi Road Shanghai 200050 People's Republic of China mma@mail.sic.ac.cn; State Key Laboratory of Molecular Engineering of Polymers, Fudan University 220 Handan Road Shanghai 200433 People's Republic of China

## Abstract

High intensity focused ultrasound (HIFU), as a promising and minimally invasive therapeutic modality against various solid tumors, has received considerable attention in the biomedical field. However, both the accuracy and efficacy of this technique are currently unsatisfactory. Herein, a nanometer-sized organic/inorganic hybrid enhancement agent for photoacoustic imaging (PAI)-guided HIFU therapy was designed and fabricated by concurrently encapsulating both Cu_2−*x*_S nanodots (NDs) and perfluorooctyl bromide (PFOB) into a poly(lactic-*co*-glycolic acid) PLGA nanocapsule (denoted CPPNs). These nanocapsules assumed a unique core/satellite/shell sandwich structure, and combined the merits of small and uniform particle size (about 120 nm), favorable biosafety, and multifunctional theranostic ability into one system. The high performance of Cu_2−*x*_S NDs in the absorption and conversion of near infrared laser confers high PAI contrast capability to the CPPNs, by which the location of the CPPNs within a tumor can be monitored successfully under PAI. Furthermore, our *in vitro* and *in vivo* results confirmed that the encapsulated PFOB in CPPNs increased the cavitation effect and thus enhanced the ablation efficacy under HIFU exposure. CPPNs show great potential as an efficient and powerful theranostic agent for future PAI-guided HIFU synergistic therapy.

## Abstract 2

A nanometer-sized inor-ganic/organic hybrid enhancement agent is constructed for photoacoustic imaging-guided high intensity focused ultrasound therapy.

## Graphical abstract

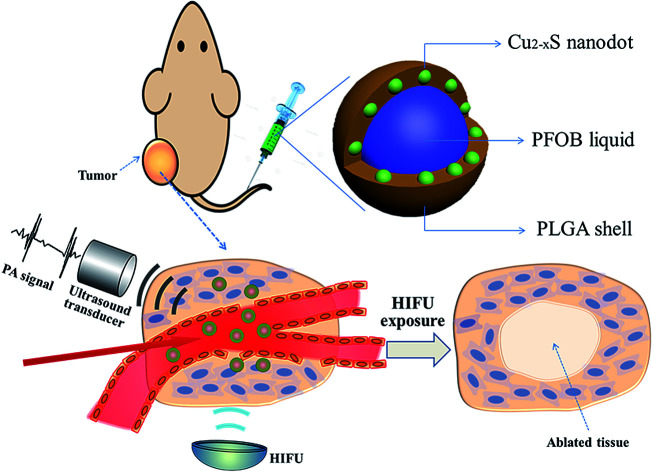

## 1. Introduction

High intensity focused ultrasound (HIFU) is considered a promising minimally invasive therapeutic modality for various solid tumors, and has been the focus of numerous studies over the past decade.^1–3^ By concentrating multiple intersecting beams of ultrasound (US) into the targeted tumor region, both hyperthermia and mechanical effects can be rapidly generated in order to efficiently induce regional coagulative necrosis and blood vessel damage.^4,5^ However, this technique still has several limitations, which restricts its broad application for clinical cancer therapy.^6,7^ The current HIFU technique generally suffers from the inevitable depth-dependent decay of acoustic energy along the US pathway, resulting in low therapeutic efficacy in treating deeply buried tumors.^8^ To solve this problem, HIFU synergistic agents (SAs), comprised of lipid or polymeric shells and fluorocarbon liquid cores, have been constructed for the enhancement of HIFU therapy by altering the acoustic environment of tumor tissues.^9–11^ Unfortunately, the large particle sizes associated with HIFU SAs, in the micrometer range resulting from the traditional emulsification fabrication method, are unfavorable for long-time blood circulation and for escaping the reticuloendothelial system, and therefore present unsatisfactory therapeutic efficacy.^12^

With a view to improving therapeutic outcomes and reducing side effects, real-time US contrast imaging is generally applied in HIFU therapy in current preclinical research, to ascertain the distribution of HIFU SAs after intravenous injection and to monitor the therapeutic response.^13^ Nevertheless, these US-guided HIFU strategies are still not satisfactory because of their inadequate therapeutic accuracy and low image contrast.^14^ In recent years, there have been several reports on HIFU SAs with magnetic resonance imaging (MRI) contrast properties, such as hollow/porous organosilica–Fe_3_O_4_ hybrid nanocapsules^15^ and manganese oxide (MnO_*x*_) nanoparticle embedded mesoporous nanoagents.^16^ These were developed with a view to enhanced MRI-guided HIFU therapy with superior imaging resolution and longer imaging duration, compared to HIFU with conventional US contrast agents. However, this type of highly integrated HIFU and MRI approach has not been widely applied in clinic due to high associated costs and complex operation.^17^ Significant advances in nanomaterial synthesis and imaging techniques are therefore urgently required to improve the therapeutic efficacy and accuracy of HIFU cancer therapy.

Photoacoustic imaging (PAI) is a noninvasive biomedical imaging technique, which combines the contrast of optical imaging and high resolution of US.^18–20^ The penetration depth of PAI in tissue-mimicking phantoms and biological tissues exceeds 5 cm, thus satisfying the imaging requirements for superficial tumors.^21^ Importantly, it has been confirmed that PAI techniques can be integrated with HIFU ablation techniques, which could help doctors to detect the location of the target tumor tissues and therefore perform HIFU ablation procedures with more accuracy.^22–24^ Moreover, to promote therapeutic efficacy, several HIFU SAs containing PA contrast agents have been successfully fabricated.^25–28^ For example, Zhang *et al.* designed, fabricated, and applied perfluorohexane-incorporated hollow mesoporous Prussian blue nanoparticles (HMPBs), which resulted in both sensitive and precise PAI and the simultaneous enhancement of HIFU therapy.^25^ Although the concept is impressive, concerns remain as to the low degradation rate and potential toxicity of HMPBs. Recently, Yan *et al.* synthesized hematoporphyrin monomethyl ether (HMME)-loaded poly(lactic-*co*-glycolic acid) (PLGA) microcapsules (HMME/PLGA) for multifunctional applications in PAI-guided HIFU treatment.^26^ In comparison to HMPB based SAs, the polymer composition endowed the system with feasible biodegradability, however, the particle size is too large (approximately 357 ± 0.72 nm in diameter) to penetrate through the barrier between vascular endothelial cells. Besides, monomeric chromophores generally have limited light absorption capacity in comparison to noble metals, thus presenting lower performance in terms of PA contrast imaging.^29^ Overall, significant structural improvements are urgently required in constructing nano-scale SAs for high-efficiency PAI-guided enhanced HIFU therapy. Recently, metal sulfide nanoparticle-based PAI contrast agents, such as copper sulfide, sliver sulfide, and bismuth sulfide nanoparticles, have received extensive attention due to their strong near-infrared absorption, unique optical properties, and well-controlled nano-scale particle size, without the need for elaborate shape design.^30–32^ Specifically and importantly, the optical absorption wavelength of Cu_2−*x*_S nanodots (NDs) can be well manipulated within a suitable optical imaging window, which is ideal for obtaining clinically relevant penetration depths with satisfactory spatial resolution. In particular, Cu_2−*x*_S NDs are highly stable *in vivo*, and can retain their photothermal and photoacoustic effects for longer than other thiolate-protected metal nanoparticles and Ag_2_S NDs.^33^

**Scheme 1:**
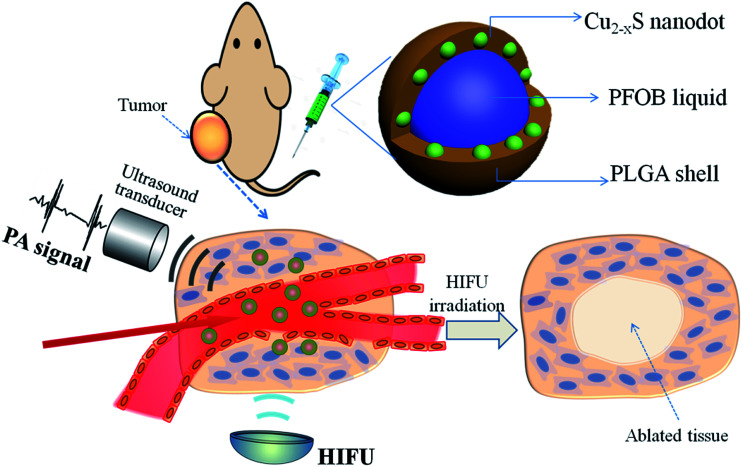
Schematic illustration of CPPNs for photoacoustic imaging-guided synergistic HIFU therapy, including the structural design and theranostic model.

With all of this in mind, herein we report on an emulsion solvent evaporation strategy to construct a multifunctional PLGA nanocapsule, concurrently encapsulating PAI contrast Cu_2−*x*_S NDs and a typical HIFU synergistic liquid, perfluorooctyl bromide (PFOB, the nanocapsules were denoted by Cu_2−*x*_S/PFOB@PLGA, CPPNs). As a promising theranostic agent, the newly designed nanocapsule system exhibited a unique core/satellite/shell sandwich structure, with PFOB liquid encapsulated in the core, as well as multiple Cu_2−*x*_S NDs sandwiched between the liquid core and polymer shell (Scheme 1). The high performance of Cu_2−*x*_S NDs in the absorption and conversion of NIR laser endows the CPPNs with high PAI contrast capability for guiding the HIFU synergistic therapy. Moreover, this organic/inorganic nanocomposite could be safely used for other potential clinical applications due to the excellent biodegradability and biocompatibility of PLGA, as well as the optimized particle size of Cu_2−*x*_S NDs, conducive to rapid elimination from the body. More importantly, the particle size of the CPPNs is only approximately 120 nm, much smaller than other HIFU therapeutic nanocapsules, rendering them more suitable for high-capacity passive accumulation within tumors. Therefore, this emerging nanoplatform should show great application potential in PAI-guided HIFU synergistic therapy, which may provide an ideal theranostic strategy for noninvasive tumor treatment in the future.

## 2. Experimental section

### 2.1 CPPN synthesis and characterization

#### 2.1.1 Preparation of Cu_2−*x*_S NDs

The preparation of Cu_2−*x*_S NDs was based on previous reports.^34,35^ Briefly, 2 mmol of sulfur was added into 12 mL of oleylamine and placed in an oil bath maintained at 70 °C. The mixture was stirred at 300 rpm until the sulfur was completely dissolved. Sequentially, 20 mL of chloroform (CHCl_3_) solution containing 3.2 mmol copper acetylacetonate and 5 mL of oleylamine were added to this solution. The resulting mixture was maintained at 70 °C until the solution became dark green after about 30 min. The obtained Cu_2−*x*_S NDs were collected by centrifugation at 10 000 rpm and washed three times with CHCl_3_, before being dispersed in CHCl_3_ and stored at 4 °C until further use.

#### 2.1.2 Preparation of CPPNs

The “emulsion evaporation” method was used to fabricate the CPPNs. Firstly, PLGA (200 mg) and PFOB (120 μL) were dissolved in 8 mL of dichloromethane (CH_2_Cl_2_), containing a designated amount of Cu_2−*x*_S NDs, by gentle stirring at 20 °C for 1 h. The CH_2_Cl_2_ mixture was then added into 40 mL of a 1.5% sodium cholate (w/v) aqueous solution. Subsequently, the mixture was emulsified using a homogenizer (HENC, Shanghai, China) and an ultrasonic probe (Bi Lang, China) at 300 W, for 1 min over ice, to obtain a light green emulsion. The emulsion was added drop-wise into 200 mL of a PVA solution (1% w/v), and placed in a temperature humidity chamber at 4 °C for 3 days. The final product was collected after centrifugation at 10 000 rpm and stored in PBS solution at 4 °C, until needed for further use.

#### 2.1.3 Material characterization

CPPN morphology was observed under transmission electron microscopy (TEM; JEOL 200CX microscope) and scanning electron microscopy (SEM; Hitachi S-4800). The elemental composition of CPPN was determined by X-ray photoelectron spectroscopy (XPS; ESCALAB 250). Ultraviolet-visible (UV-vis) absorption spectra were obtained on a Shimadzu UV-3101PC spectrometer. Fourier transform infrared spectroscopy (FTIR) spectra were recorded on a Nicolet IS10 instrument. Thermo-gravimetric (TG) analysis was carried out by a NETZSCH STA 449C system. Nanoparticle size distribution was measured by dynamic light scattering (DLS). ^19^F NMR spectra were captured by a Bruker Avance AV 300 NMR spectrometer (300 MHz). The element concentration in aqueous solution was measured using inductively coupled plasma mass spectrometry (ICP-MS, Agilent 7500ce).

### 2.2 Cu_2−*x*_S NDs stability evaluation

2 mg of CPPNs were transferred into dialysis bags (cut off molecular weight 3500 g mol^−1^). Next, two bags were soaked in plastic tubes containing 30 mL saline at pH 7.4 and 6.0, separately. The tubes were placed into a shaking table at 150 rpm and 37 °C. At various time intervals, 2.0 mL of the solution was collected and diluted with deionized water. Then, the copper ion concentration in solution was measured through ICP-MS.

### 2.3 CPPN biosafety evaluation

#### 2.3.1 Ethical statement of animal experiment

The study protocol was approved by the Animal Care Ethics Commission of Shanghai Tenth People's Hospital, Tongji University School of Medicine. All animal experiments were performed in compliance with relevant laws and institutional guidelines of the National Institute of Health.

#### 2.3.2 CPPN *in vitro* cytotoxicity

The mammary carcinoma 4T1 cell line was cultured in DMEM medium with 10% fetal bovine serum (FBS) and 1% penicillin–streptomycin. TEM was used to evaluate nanoparticle internalization in 4T1 cells which were treated with 100 μg mL^−1^ of CPPNs for 4 h. A cell counting kit-8 (CCK8) was used to evaluate the cytotoxicity of the CPPNs. Three thousand cells per well were incubated in a 96-well plate at 37 °C in a humidified 5% CO_2_ incubator for 24 h. Then, the medium was replaced by fresh medium containing CPPNs at concentrations of 0, 3.07, 6.13, 12.5, 25, 50, 100, and 200 μg mL^−1^. After incubation for another 24 h, 10 μL of CCK8 solution was added into each well. The absorbance of each well was read at 450 nm after 1 h by a microplate reader.

#### 2.3.3 Hematological, biochemical and pathological analyses

Eighteen Balb/c nude mice (body weight: ∼25 g) were divided into three groups (*n* = 6): (I) tail vein injection of 0.1 mL saline; (II) tail vein injection of 0.1 mL saline containing CPPNs at a dose of 100 mg kg^−1^; (III) tail vein injection of 0.1 mL saline containing CPPNs at a dose of 200 mg kg^−1^. All of the nude mice were sacrificed 28 days after the intravenous injection. Whole blood and serum were collected for hematological analyses to assess biochemical parameters, including white blood cell (WBC) count, red blood cell (RBC) count, blood urea nitrogen (BUN), creatinine (Cr), and so on. Meanwhile, the main organs, including the heart, liver, spleen, lung, and kidney, were resected and fixed in 10% formalin. Hematoxylin and eosin (H&E) staining was performed to assess differences in organ pathology between each group.

### 2.4 Establishment of the tumor-bearing nude mice model and bio-distribution of CPPNs

Approximately 6 × 10^7^ 4T1 cells were injected into the back of each nude mouse with a volume of 0.15 mL. Tumor dimensions were measured daily using a caliper and tumor volume was calculated according to the following equation:

1 *V* = π × *L* × *W*^2^/6,

where *V* represents tumor volume, *L* tumor length, and *W* denotes the tumor width.

When the volume of the tumor reached ∼1 cm^3^, six tumor-bearing nude mice were randomly divided into two groups to evaluate the CPPN bio-distribution, 1 h and 24 h after the intravenous injection. The mice were sacrificed at these time points, and Cu concentrations in different organs (tumor, heart, liver, spleen, lung, and kidney) were assessed through ICP-MS after digestion by HClO_4_/HNO_3_ (v/v = 3/1) solution.

### 2.5 Evaluation of CPPN performance in PAI

#### 2.5.1 *In vitro* PAI

CPPN suspensions were placed in plastic tubes at final Cu concentrations of 70, 140, and 280 μg mL^−1^. The photoacoustic (PA) average amplitude of saline was measured as a baseline reference. Then, the suspension PA images and the corresponding PA average amplitudes were recorded by a VEVO LASR imaging system (FUJIFILM Visual Sonics, Inc., USA).

#### 2.5.2 *In vivo* PAI

A tumor-bearing mice model was used to evaluate the *in vivo* PAI capacity of the CPPNs. Each mouse was anesthetized by inhaling sevoflurane and fixed under an US probe. PA images of the tumor region at a wavelength of 930 nm were collected before and 1 h after intravenous injection of 0.1 mL saline containing CPPNs with a Cu concentration of 70 μg mL^−1^.

### 2.6 Evaluation of CPPNs in HIFU ablation

#### 2.6.1 *In vitro* evaluation of CPPN-HIFU effects

A JC200 HIFU treatment system (Chongqing HIFU Medical Technology Limited-liability Company, China) was employed to investigate the influence of CPPNs on HIFU ablation. Briefly, 2 mL of a CPPN suspension with a particle concentration of 2.5 mg mL^−1^ was placed in an Eppendorf (EP) tube and was subjected to HIFU irradiation under an output power of 150 W for 10 s (5 s twice, 5 s intervals). EP tubes filled with saline were used as controls and received HIFU irradiation under the same conditions. B-mode US images and their corresponding grey scale values (measured by GrayVal 1.0 software) were recorded before and after HIFU ablation. CPPN morphological changes were observed under SEM.

#### 2.6.2 *Ex vivo* evaluation of HIFU effect

Degassed bovine livers were used to evaluate HIFU enhancement by CPPN inclusion. After each liver was placed into a tank filled with degassed water, 200 μL of saline (controls) or CPPN suspensions, at a concentration of 2.5 mg mL^−1^, were injected into focused regions. They were then subjected to single irradiation HIFU treatments with output acoustic powers of 80 or 140 W for 10 s. The corresponding grey scale values before and after HIFU ablation were recorded. The coagulative necrosis volume of the liver sample in each group was measured and calculated according to eqn (1). Parallel experiments were repeated three times for each sample to obtain an average necrosis volume value.

#### 2.6.3 *In vivo* evaluation of HIFU outcomes

*In vivo* HIFU ablation efficacy was evaluated when the tumor volume reached approximately 1 cm^3^. The tumor-bearing mice were anesthetized by inhaling sevoflurane, and were subjected to HIFU exposure at an acoustic power output of 140 W for 10 s (5 s twice, 5 s interval) after the administration of one of the following: (A) intravenous injection with saline; (B) intravenous injection of a 2.5 mg mL^−1^ CPPN suspension; (C) intravenous injection of a 5.0 mg mL^−1^ CPPN suspension. The mice were euthanized 1 h after receiving HIFU treatment. The tumor from each mouse was removed and cut into two parts of similar volume. One part of the tumor was stained with triphenyl tetrazolium chloride solution (TTC, 1 wt%) for about 15 min, to define the boundary between the coagulative necrotic area and the surrounding tissue. Then, the ablation volume was measured and calculated according to eqn (1). Meanwhile, the other section was fixed in 10% formalin for further pathological analyses, including H&E staining, terminal deoxynucleotidyl transferase-mediated dUTP nick-end labeling (TUNEL), immunohistochemical staining with antibodies against the proliferating cell nuclear antigen (PCNA), and Bio-TEM (the experimental procedures are detailed in the ESI†). Furthermore, the apoptotic index (AI) and proliferative index (PI) were calculated from the ratio of positively stained tumor cells to all cells by selecting six random field from microscopy images captured at 400× magnification.

## 3. Results and discussion

### 3.1 Structural characterization of Cu_2−*x*_S/PLGA nanocapsules

**Fig. 1:**
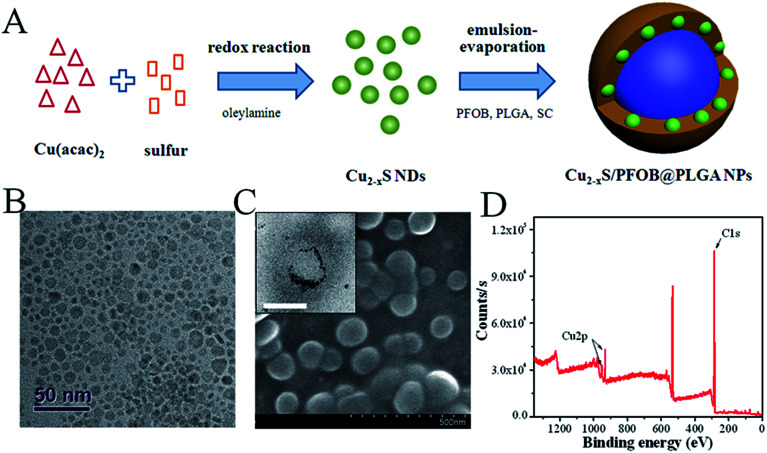
(A) Schematic diagram of the CPPN synthetic procedure (B) TEM images of Cu_2−*x*_S NDs. (C) SEM and TEM (inset) images of CPPNs. The scale bar of the inset TEM image is 50 nm. (D) XPS analysis of CPPNs. The Cu 2p spectrum was further divided into Cu 2p_1/2_ and Cu 2p_3/2_, and the corresponding peaks were measured at 952.4 eV and 932.5 eV, respectively (Fig. S3†).

The fabrication of CPPNs is schematically illustrated in Fig. 1A. Hydrophobic oleylamine-coated Cu_2−*x*_S NDs were firstly synthesized in oleylamine solution through a low-temperature redox reaction. TEM images show that Cu_2−*x*_S ND particle sizes ranged from 2.5 to 10.5 nm, with an average diameter of 5.9 nm (Fig. 1B and S1†). Such small dimensions are considered bio-safe for clinical cancer therapy translation since they are easily removed from blood by renal clearance, more so than larger analogues.^34^ A typical emulsion-evaporation process in a mixed dichloromethane/water solvent was employed to simultaneously encapsulate the fabricated Cu_2−*x*_S NDs and super-hydrophobic PFOB HIFU-synergistic liquid into the PLGA capsule. Dichloromethane was chosen as the nonpolar liquid phase to ensure full solubility of PLGA and PFOB, as well as the Cu_2−*x*_S NDs. Sodium cholate was introduced into the aqueous phase in the synthetic processing of Cu_2−*x*_S/PFOB@PLGA nanoparticles, distinct from a traditional emulsion-evaporation strategy, in order to shrink the diameters of the PLGA-based capsules by decreasing oil/water interfacial tension.

The spherical morphology and microstructure of the CPPNs are shown in Fig. 1C. Notably, a considerable quantity of NDs with deep contrast were uniformly distributed around the PFOB liquid inner core, which corresponded to the Cu_2−*x*_S NDs, as evidenced by the XPS results detailed in Fig. 1D and S2.† The nanocapsule Cu 2p spectrum exhibited two sets of peaks due to differential elemental valencies. Correspondingly, the characteristic binding energies seen at 932.6 and 952.5 eV were assigned to Cu^+^, while the peaks observed at 933.7 and 954.6 eV were attributed to Cu^2+^ (Fig. S3†).

**Fig. 2:**
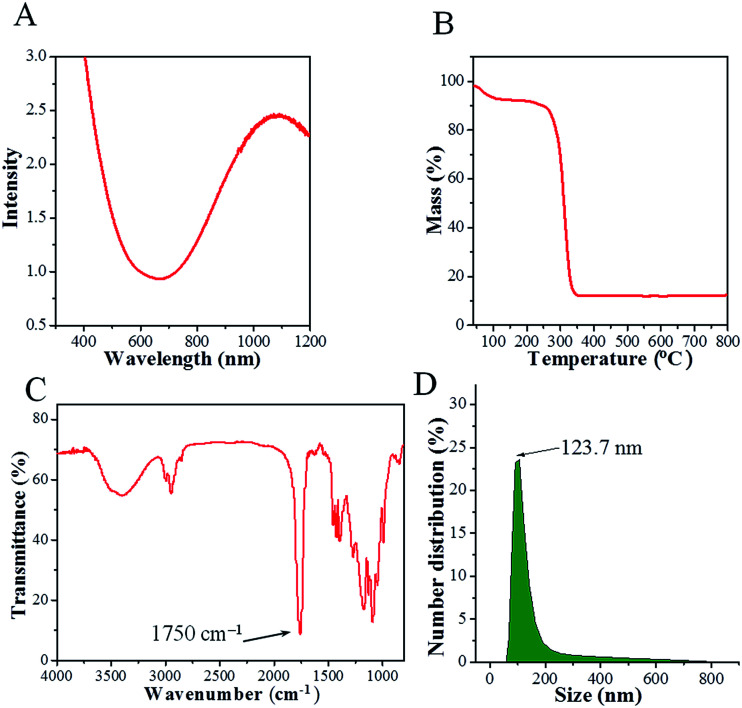
(A) UV-vis absorbance spectra of CPPNs in aqueous solution. (B) TG curve of CPPNs. (C) FTIR spectrum of CPPNs. (D) The hydrodynamic diameter distribution of CPPNs in saline solution, as measured by DLS.

The UV-vis spectrum of a CPPN aqueous dispersion is presented in Fig. 2A. High optical absorption can be observed over the 600 to 1100 nm wavelength range, which is highly consistent with the spectrum of pure Cu_2−*x*_S NDs in dichloromethane (shown in Fig. S4†), indicating that the PLGA coating had no influence on the optical properties of Cu_2−*x*_S NDs. Moreover, such strong optical absorption in the near-infrared region points to the potential of CPPNs as contrast agents for *in vivo* PAI. Additionally, the release of copper ions at different time points under two pH conditions (7.4 and 6.0) was determined to evaluate the stability of the Cu_2−*x*_S NDs. The results (Fig. S5†) revealed that the quantity of copper ions released was less than 0.1% for both pH conditions, indicating that Cu_2−*x*_S NDs can retain their photoacoustic effects for long periods.

The organic composition of the CPPNs was elucidated through TG and FTIR analyses. The mass percentage of Cu_2−*x*_S ND was about 10% (w/w) of the whole CPPN, as determined by TG analysis (Fig. 2B). The FTIR spectrum of the CPPNs is presented in Fig. 2C. A sharp absorption band at about 1750 cm^−1^ can be observed, characteristic of carbonyl stretching vibrations, which are present in both the d,l-lactide and glycolide monomer components of PLGA. The strong bands observed between 1300 cm^−1^ and 1050 cm^−1^ correspond to both asymmetric and symmetric C–C(=O)–O vibrations. Additionally, there were also obvious bands observed between 3000 cm^−1^ and 2800 cm^−1^, due to aliphatic C–H stretching vibrations, present over all of the polymer skeleton. Notably, characteristic sodium cholate fingerprint peaks (1564 cm^−1^ and 1649 cm^−1^) were absent, indicating that sodium cholate was not retained within the CPPNs.^36^ In summary, the CPPN FTIR spectrum was very similar to the standard PLGA spectrum described in the reported literature, confirming that the organic composition of the capsule shell was mostly comprised of PLGA.^37^ Furthermore, ^19^F NMR was used to confirm the existence of PFOB liquid in the CPPNs. The spectra (Fig. S6†) showed obvious characteristic peaks of PFOB, confirming the successful encapsulation of PFOB within the PLGA nanoparticles.

The CPPNs were uniform in size with an average hydrodynamic size of 123.7 nm and 145.8 nm at room temperature and 37 °C, respectively, as evaluated by DLS technique (Fig. 2D). Comparatively, the hydrodynamic size of CPPNs at 37 °C was somewhat higher than the same at room temperature, which can be attributed to the swelling behavior of the PLGA polymer and the PFOB liquid core. Additionally, the stability of CPPNs was evaluated by suspending the particles in saline at 37 °C. Notably, the mean hydrodynamic size of the CPPNs remained within a 140 nm to 180 nm range, confirming the stability of the CPPNs (Fig. S7†). Importantly, the vast majority of CPPNs exhibited nanometer grade dimensions, which is much smaller than the Bi_2_S_3_-embedded PLGA nanocapsules (Bi_2_S_3_/PLGA) and HMME/PLGA microcapsules mentioned and reported previously.^9,26^ It is believed that such small sized CPPN particles (∼123.7 nm) would be conducive to crossing the vascular endothelium and accumulating within tumors through the enhanced permeability and retention (EPR) effect.^38^

### 3.2 CPPN biosafety

**Fig. 3:**
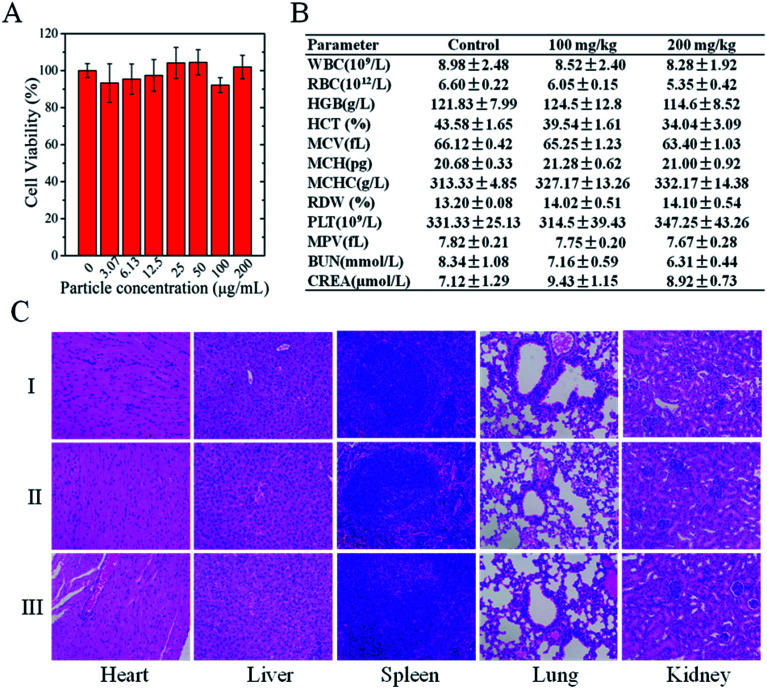
(A) Cell viability of 4T1 cells treated with different CPPN concentrations (0, 3.07, 6.13, 12.5, 25.0, 50.0, 100.0, and 200.0 μg mL^−1^) for 24 h. (B) Hematological and blood biochemical analyses of mice 28 days after receiving an intravenous injection of saline (control) or CPPNs. (C) H&E staining of organ sections collected from different groups of mice 28 days after receiving an intravenous injection of saline (control) or CPPNs.

We evaluated the biosafety of CPPNs through *in vitro* cytotoxicity analyses and *in vivo* toxicity tests in healthy nude mice. TEM images show that CPPNs could be efficiently internalized after incubation with 4T1 cells for 4 h (Fig. S8†). The CCK-8 assay was employed to further confirm the effect of CPPNs on cell viability, as shown in Fig. 3A. After culture with different concentrations of CPPNs for 24 h, there was no obvious difference in the cell proliferation amongst the groups, which points to favorable CPPN biocompatibility.

Four weeks after receiving a tail vein injection of CPPN, negligible influence on hemocompatibility was observed in the biochemical and complete blood analysis of nude mice (Fig. 3B). Furthermore, there was no obvious histopathologic change or damage to organs, including heart, liver, spleen, lung, and kidney, in either the control or the test groups, as shown in Fig. 3C. These *in vivo* and *in vitro* results demonstrate the favorable biosafety of CPPNs in physiological environments.

### 3.3 CPPNs enhance PAI quality

**Fig. 4:**
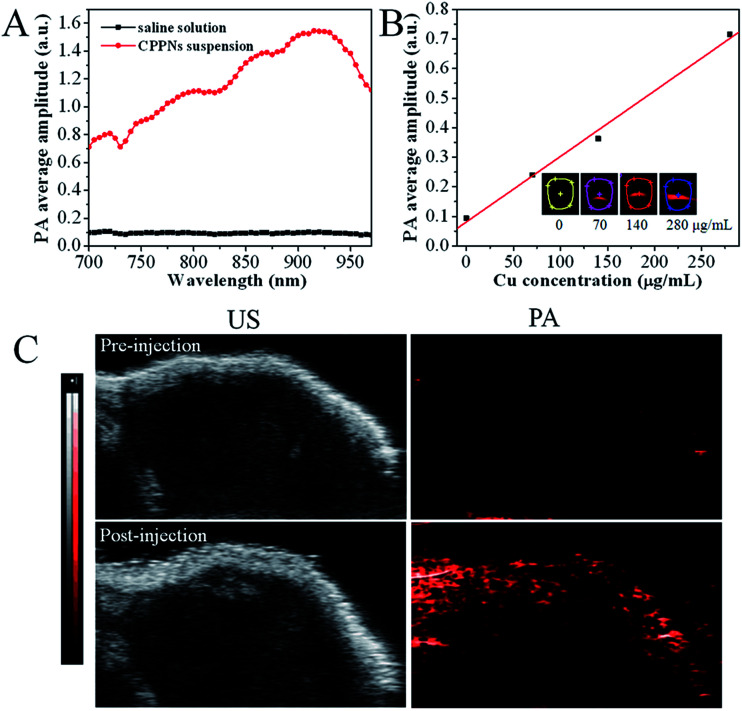
(A) The CPPN suspension PA signals over the excitation range of 700 nm to 970 nm. (B) The CPPN PA average amplitude at different concentrations (0, 70, 140, and 280 μg mL^−1^). (C) The B-Mode ultrasound and PA images of tumors before and 1 h after an intravenous injection of CPPN suspension into tumor-bearing mice.

In order to investigate the influence of CPPNs on PAI, both *in vitro* and *in vivo* imaging assessments were conducted using a VEVO LASR imaging system, with adjustable excitation wavelength in the near-infrared range. Fig. 4A shows the PA response of CPPNs in saline solution over an excitation range of 700 to 970 nm. The PA signal baseline was approximately 0.1, in contrast, obvious enhancement of the PA response could be observed with the addition of CPPNs, probably due to the optical absorption and thermophysical properties of Cu_2−*x*_S NDs. Notably, the fact that the CPPN enhancement effect was not uniform over the entire spectral range demonstrates that the optical and thermophysical properties were selectively sensitive across the near-infrared region. The strongest PA response was at a wavelength of 930 nm, which was chosen as the imaging system excitation wavelength for subsequent experiments, based on this result. Additionally, PA response enhancement increased with increasing CPPN concentration, Fig. 4B. Subsequently, in order to confirm the efficacy of CPPNs in PAI *in vivo*, 4T1 tumor-bearing Balb/c nude mice were injected intravenously with 0.1 mL of saline, as controls, or CPPNs at a concentration of 200 μg mL^−1^. Tumor-site B-mode US images and PA images at a wavelength of 930 nm were captured before and after the administration of CPPNs. As shown in Fig. 4C, the PA signal could not be detected before injection. In contrast, obvious PA signal enhancement in the tumor region could be observed 1 h post CPPN injection. Overall, the above results demonstrate that CPPNs hold significant potential as efficient PA contrast agents, both *in vitro* and *in vivo*.

Furthermore, ICP-MS was employed to quantify the distribution of CPPNs within tumors and typical organs (heart, liver, spleen, lung, and kidney) 1 h and 24 h after intravenous CPPN injection. The statistical results show that about 7.1% of Cu had been uptaken by tumor tissues 1 h post-injection, indicating that enhanced PAI can be mainly attributed to tumor-site enrichment of Cu_2−*x*_S NDs (Fig. S9†). Notably, the Cu content had decreased to 2.2% in the tumor region after 24 h, which suggested that PLGA-coated Cu_2−*x*_S NDs were gradually eliminated from the tumor, probably due to both PLGA degradation and the small particle dimensions of the Cu_2−*x*_S NDs, which favors physiological elimination.

### 3.4 *In vitro* HIFU treatment of CPPNs

**Fig. 5:**
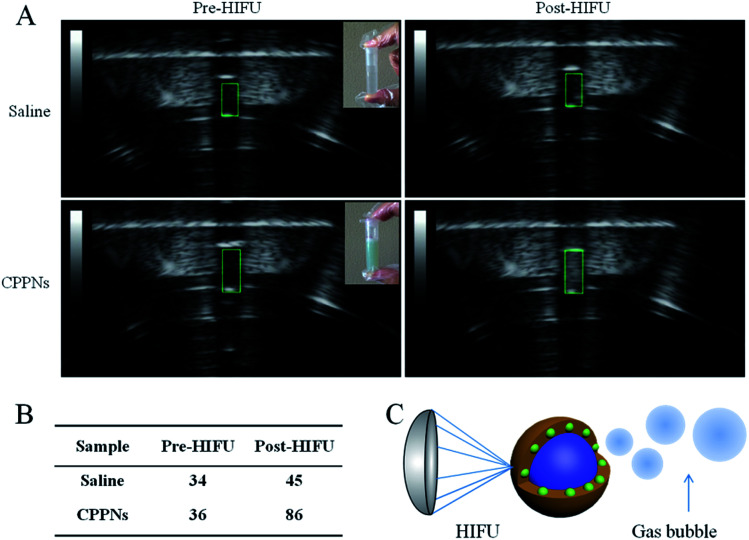
(A) The B-mode ultrasound imaging shows changes in echogenicity before and after HIFU irradiation in the saline group and the CPPN group. (B) The corresponding gray scale of each group. (C) Schematic illustration of PFOB microbubble formation under HIFU irradiation.

PFOB, which was sealed *in situ* as the inner core of the CPPNs, exhibits distinct characteristics such as relatively low boiling point and superhydrophobicity, and is considered an excellent HIFU synergistic liquid candidate. To confirm that HIFU was able to trigger the “liquid to gas” phase transition of the encapsulated PFOB, B-mode US images of a CPPN suspension were recorded before and post HIFU exposure. Saline solutions were subjected to HIFU exposure under the same conditions for comparison. Variations of echogenicity in different groups could be immediately observed after receiving HIFU irradiation. As shown in Fig. 5, there was no obvious gray scale difference in the saline group before and after HIFU irradiation, with corresponding gray scale values of 34 dB to 45 dB, respectively. In stark contrast, high echogenicity with a sharp gray scale increase from 36 dB to 86 dB was observed for the CPPN group, pre- and post-irradiation, respectively: indicative of microbubble formation due to the HIFU-induced temperature rise. Morphological CPPN changes were also observed under SEM (Fig. S10†) after HIFU irradiation. Interestingly, the nanometer-size spherical shape of the CPPNs (shown in Fig. 1C) could hardly be observed in the SEM images. Instead, large irregular granules with micrometer dimensions could be observed, indicating structural collapse of the polymer shell. This phenomenon indirectly suggests that HIFU irradiation may induce PFOB release based on a phase transition route. Importantly, according to previous studies, it is believed that these PFOB gas microbubbles may amplify the cavitation effect, resulting in HIFU synergistic effects.^39^

### 3.5 *Ex vivo* HIFU treatment of CPPNs

**Fig. 6:**
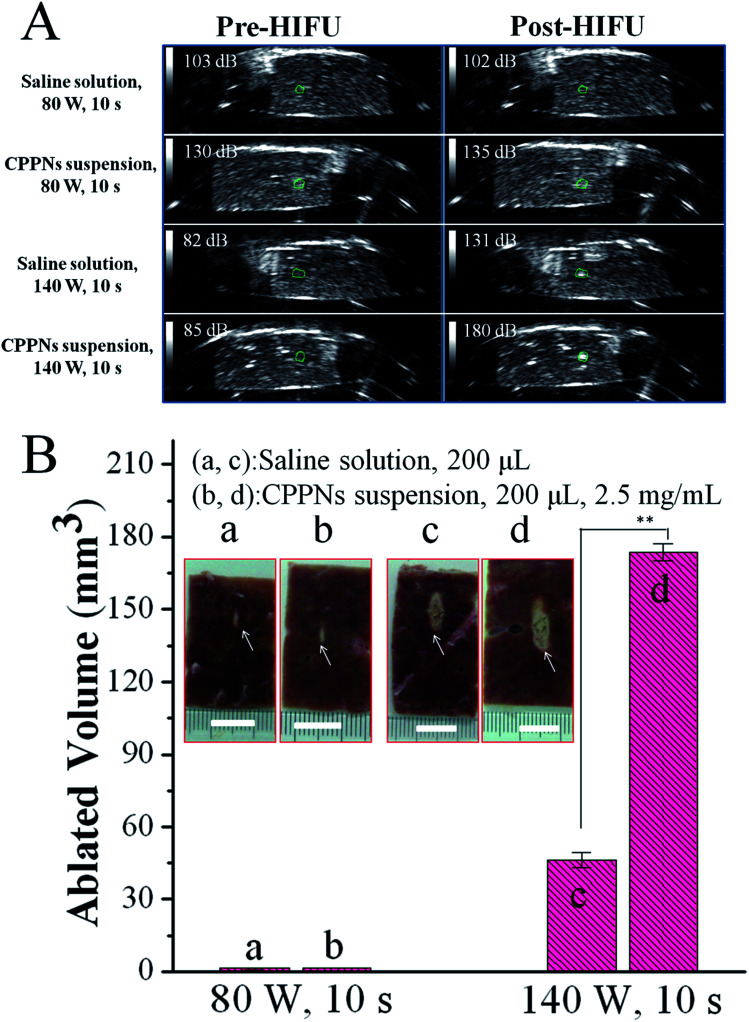
(A) Ultrasound images of the focus site (shown in the green circle) in *ex vivo* degassed bovine liver, before and after HIFU irradiation for 10 s with 200 μL of saline or CPPN suspension at a concentration of 2.5 mg mL^−1^. Average gray-scale value of the focused site is indicated at the top left of each image. (B) The pictures of degassed bovine liver samples before and after HIFU treated with saline (a, c) and CPPNs (b, d) at an output of 80 W and 140 W. The histogram shows the measured coagulative necrotic volume in each group.

In order to assess the acoustic enhancement capability of CPPNs *ex vivo*, B-mode US imaging was employed to observe the gray scale changes in degassed bovine livers at a HIFU focus point. This allowed for the quantitative determination of acoustic environmental changes owing to coagulative ablation. In detail, 200 μL of saline or CPPN solution (2.5 mg mL^−1^) was injected into a focused region of degassed bovine tissue under the guidance of US imaging, which was then subjected to HIFU at an output power of 80 W or 140 W for 10 s. As shown in Fig. 6A, there were no distinguishable gray scale changes in the saline or CPPN groups when the degassed bovine livers were exposed to HIFU at a power output of 80 W for 10 s. When the power was increased to 140 W, the saline group presented obvious enhancement in echogenicity, with an average gray scale change from 82 dB to 131 dB. Impressively, a sharp increase in gray scale, from 85 dB to 180 dB, was observed for the CPPN group, a much larger change than for the saline group.

The volume of coagulative necrotic tissue in each degassed bovine liver was also calculated after HIFU exposure. As shown in Fig. 6B, no visual difference could be found between the saline group and the CPPN group when the output power was fixed at 80 W. This result is consistent with previous studies that have demonstrated that relatively low HIFU output power fails to successfully trigger the PFOB cavitation effect, due to low thermal accumulation by HIFU exposure under those conditions.^40^ As expected, significant necrotic volume enlargement was observed in the CPPN group (173.6 mm^3^) compared to the saline group (46.3 mm^3^) when the power was increased to 140 W, indicating the excellent enhancement influence of CPPNs at relatively high HIFU output power.

### 3.6 *In vivo* HIFU treatment of CPPNs

**Fig. 7:**
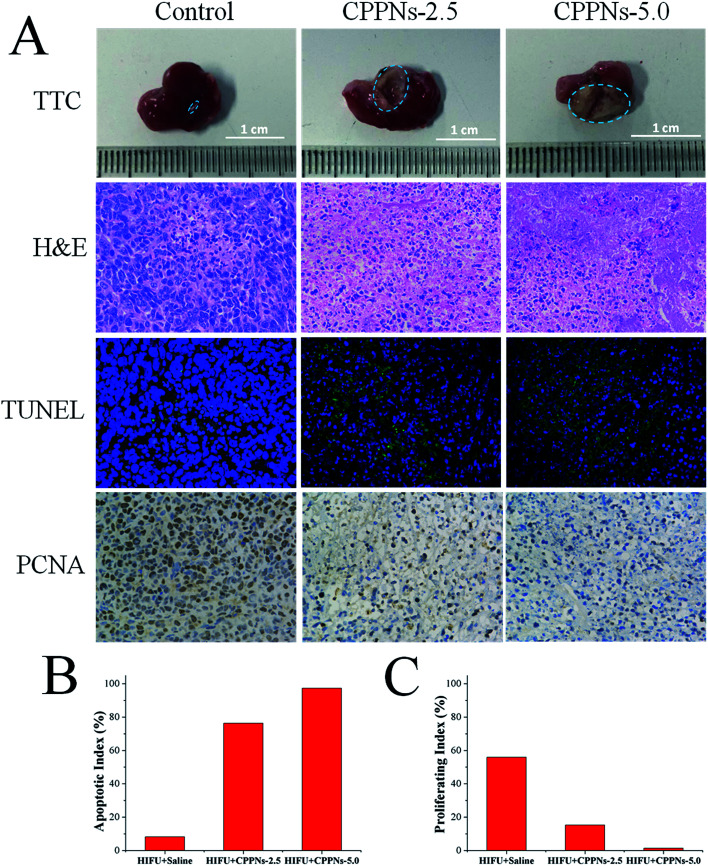
(A) TTC, H&E staining, TUNEL and immunohistochemical staining for PCNA after treatment with saline control, CPPNs-2.5 and CPPNs-5.0, with HIFU irradiation at an output of 150 W for 10 s (5 s × 2 times, 5 s interval). (B) The corresponding apoptotic index (AI) and (C) the proliferative index (PI) of the tumor tissues in the control group, CPPNs-2.5 group and CPPNs-5.0 group.

To investigate the therapeutic efficacy of HIFU treatment *in vivo*, 4T1 tumor-bearing mice were randomly divided into three groups, and received intravenous injection of different samples of equivalent volume (0.25 mL): (A) saline (control); (B) 2.5 mg mL^−1^ CPPNs (defined as CPPNs-2.5) and (C) 5.0 mg mL^−1^ CPPNs (defined as CPPNs-5.0). For each group, HIFU exposure was conducted at an output power of 150 W for 10 s (5 s twice, 5 s interval) 1 h after intravenous injection. As shown in Fig. 7A, a remarkable demarcated region between the coagulative necrotic region (white) and non-ablated tissues (red) could be observed in the gross inspection cross section of the CPPN-treated groups after TTC staining. In particular, the tumor was almost completely ablated when the CPPN concentration was increased to 5.0 mg mL^−1^. The quantitative necrotic volumes of the CPPNs-2.5 and CPPNs-5.0 groups were measured to be 108.2 mm^3^ and 136.1 mm^3^, respectively, which were significantly larger than that of the control group (2.5 mm^3^).

To further confirm the degree of destruction after HIFU irradiation in all groups, a series of pathological examinations were undertaken, including H&E staining, TUNEL, and immunohistochemical marking for PCNA. The H&E staining showed that there was no distinct cell damage in the control group, indicating the low therapeutic efficiency of HIFU irradiation alone in the tumor xenograft model. In contrast, obvious coagulative necrosis characteristics, including karyorrhexis, karyopyknosis, and nucleolysis, were observed in both CPPNs-treated groups.

Additionally, the TUNEL assay was applied to evaluate cell apoptosis. The degree of cell apoptosis was reflected by the intensity of the green fluorescence signal, which represented the quantity of single strands of broken DNA in damaged cells. As shown in Fig. 7A, apoptosis was significantly more prevalent in the two CPPN-treated groups than the control group. Quantitatively, the AI of each group was calculated and the results are detailed in Fig. 7B. The two CPPN groups had much higher AI values (76.32% and 97.33% for CPPNs-2.5 and CPPNs-5.0, respectively) than the control group (8.22%), further indicating that CPPNs can improve the therapeutic efficacy of HIFU treatment by inducing tumor cell apoptosis.

The expression of PCNA in each group was also assessed by immunohistochemical staining, which reflects tumor cell proliferation ability. It was easy to find the PCNA-positive tumor cells in the control group, with brown granules observable in the cell nuclei; implying incomplete ablation of tumor tissue (Fig. 7A). However, PCNA-positive tumor cells could hardly be found in the CPPN-treated groups. As shown in Fig. 7C, the corresponding PI of the control group (55.97%) was much higher than those of the CPPN groups (15.26% and 1.40% for CPPNs-2.5 and CPPNs-5.0, respectively). These results suggest that the expression of PCNA was significantly suppressed after HIFU irradiation in the presence of CPPNs.

Finally, tumor cell morphology in each group was observed under TEM (Fig. S11†). While cells in the control group mostly retained their morphological character, the cells in the CPPN groups presented irreversible cell membrane damage and lost their nuclear and mitochondrial integrity. Overall, both the *ex vivo* and *in vivo* results demonstrate that CPPNs show excellent synergistic capability in HIFU irradiation against tumor tissues.

## 4. Conclusions

In summary, an organic/inorganic hybrid enhancement agent for PAI-guided HIFU therapy was successfully fabricated by an emulsion-solvent evaporation strategy. The synthesized CPPNs combined the merits of small particle size (∼123.7 nm), favorable biosafety, and multifunctional theranostic ability into an integrated system. More importantly, the CPPNs exhibited high performance ability in PA contrast imaging as well as in the enhancement of HIFU tumor treatment, both *in vitro* and *in vivo*. Overall, we believe that the CPPNs are promising theranostic agents for PAI-guided HIFU synergistic therapy, which have great potential for future clinical cancer therapy applications.

## Conflicts of interest

There are no conflicts to declare.

## Supplementary Material

RA-008-C7RA12074E-s001
