# Supplementary material for: Small PLGA nanocapsules Co-encapsulating copper sulfide nanodots and fluorocarbon compound for photoacoustic imaging-guided HIFU synergistic therapy
Source: RSC Adv. 2018 Jan 24;8(9):4514–24. doi: 10.1039/c7ra12074e (PMC9077886; doi:10.1039/c7ra12074e)
Supplement: RA-008-C7RA12074E-s001 [file RA-008-C7RA12074E-s001.pdf]

Electronic Supplementary Material (ESI) for RSC Advances.
This journal is © The Royal Society of Chemistry 2018

Supplementary Data

1. Materials

Copper acetylacetonate, sulfur, lactic-co-glycolic acid (PLGA, MW=30-70 kDa, 89% hydrolyzed), perfluorooctyl bromide (PFOB), and oleylamine were purchased from Sigma Aldrich. Chloroform and dichloromethane were purchased from Lingfeng Company. The 4T1 cell line, penicillin-streptomycin (1%), and the Cell Counting kit-8 (CCK8) were purchased from Jrdun Biotechnology. The medium DMEM was purchased from HyClone (US). Fetal Bovine Serum (FBS; 10%) was purchased from PAA (German). Balb/c nude mice were purchased from Shanghai Laboratory Animal Co. Ltd. (SLAC, Shanghai, China) and raised at the Laboratory Animal Centre, Shanghai Tenth People's Hospital of Tongji University, China.

2. Supplementary experimental section

2.1 Terminal deoxynucleotidyl transferase-mediated dUTP-biotin nick ending-labeling method (TUNEL) protocol

Cell apoptosis was evaluated by the TUNEL method according to the manufacturer's protocol. Briefly, paraffin sections were deparaffinized and rehydrated by xylene and ethanol at different concentrations, respectively. The slides were treated with proteinase K (pH 7.4) for 30 min at 37 ˚C and rinsed three times. Then, Tris was added to cover the tissue and after 20 min incubation, the section was rinsed with phosphate-buffered saline (PBS) three times for a total of 15 min. Subsequently, reagents 1 and 2 from the TUNEL kit (1:29, Roche) were added into the sections before incubation in a wet box for 2 h in a water bath. Finally, 4',6-diamidino-2-phenylindole (DAPI) was used to stain the cell nucleii and anti-quenching agent was added. The slides were visualized using a fluorescence microscope. The apoptosis index (AI) was defined as the ratio of apoptosis-positive tumor cells to all cells and was calculated by counting cells in six random areas at 400x magnification under an optical microscope.

2.2 PCNA Immunohistochemistry

The slides were deparaffinized through xylene, and a 100%, 95%, 90%, 80%, and 70% ethyl alcohol series and then rinsed in water. Antigen retrieval was performed by microwave-heating in modified citrate buffer (1 L, pH 6.0). A solution of 0.3% hydrogen peroxidase in methanol was used to quench endogenous peroxidase activity for 20 min. PCNA-positive cells were confirmed from 6 random fields at 400x magnification under an optical microscope. The positive index (PI) was defined as the ratio of positively stained tumor cells to all cells and was calculated by counting 6 random areas at 400x magnification under an optical microscope.

3. Supplementary figures

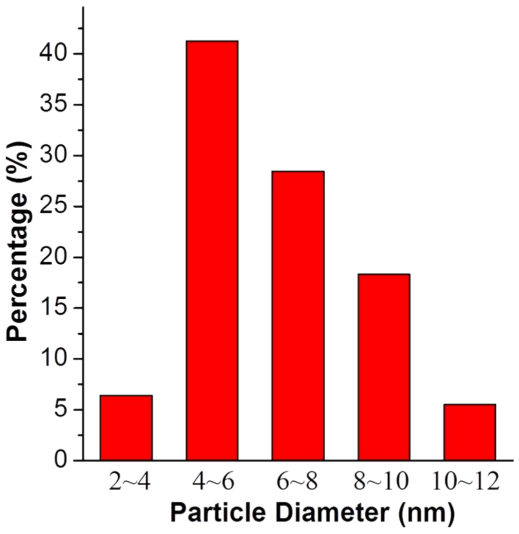

Fig. S1: The particle size distribution shows that approximately 94.5% of the $Cu_{2-x}S$ NDs were less than 10 nm in size.

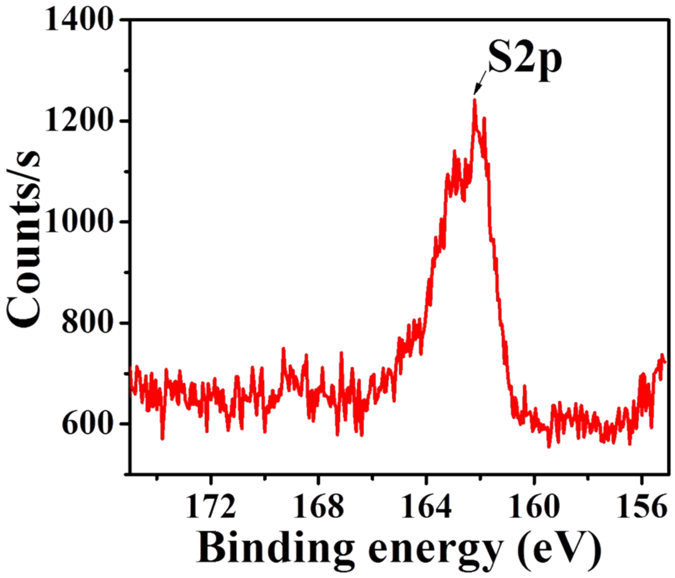

Fig. S2: XPS spectrum of CPPNs confirmed the existence of sulfur.

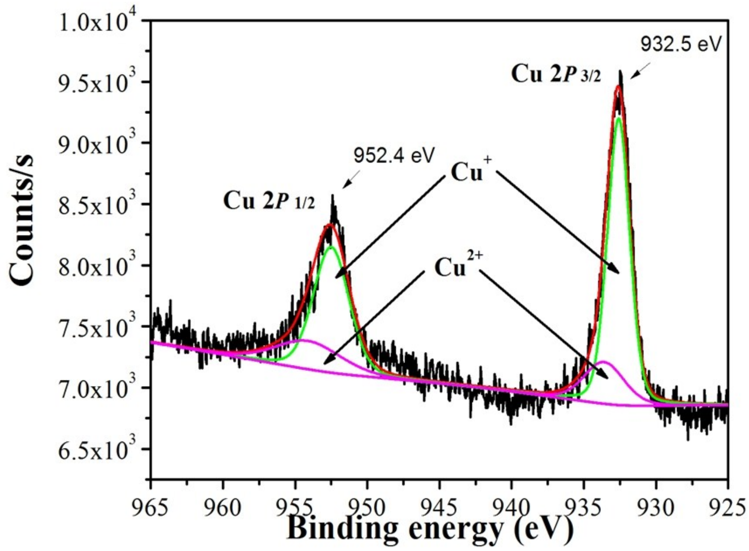

Fig. S3: XPS spectrum of CPPNs shows two peaks that can be assigned to Cu.

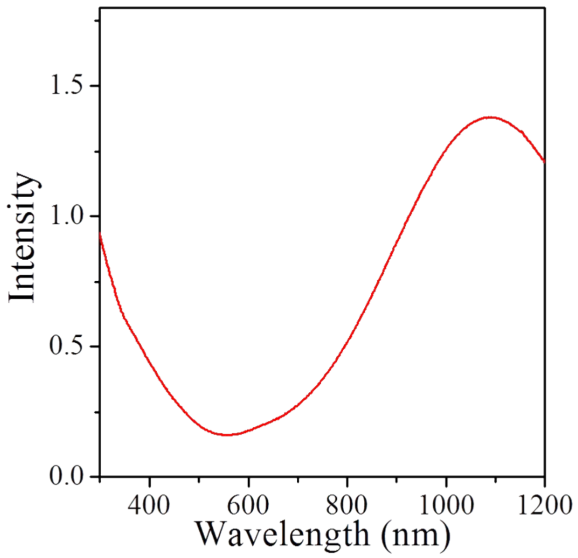

Fig. S4: UV-vis spectrum of $Cu_{2-x}S$ NDs dispersed in chloroform.

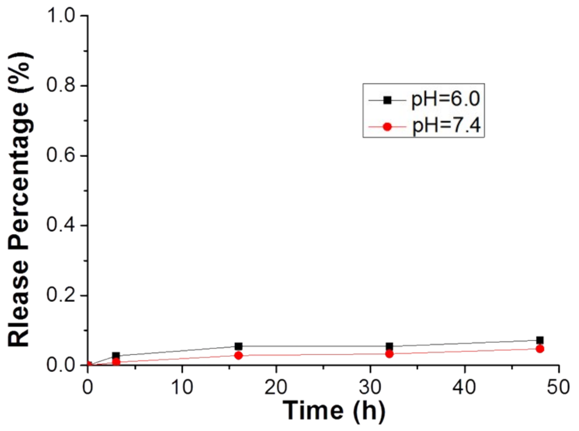

Fig. S5: Copper ions released over time at pH 7.4 and 6.0. The percentage of copper ions released was less than 0.1% in both conditions, indicating that the $Cu_{2-x}S$ NDs could retain their photoacoustic properties over extended periods of time.

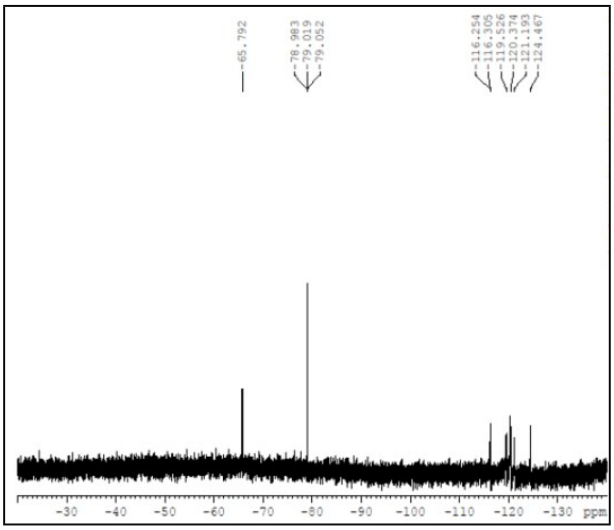

Fig. S6: ^{19}F-NMR spectrum of PFOB liquid encapsulated within the CPPNs.

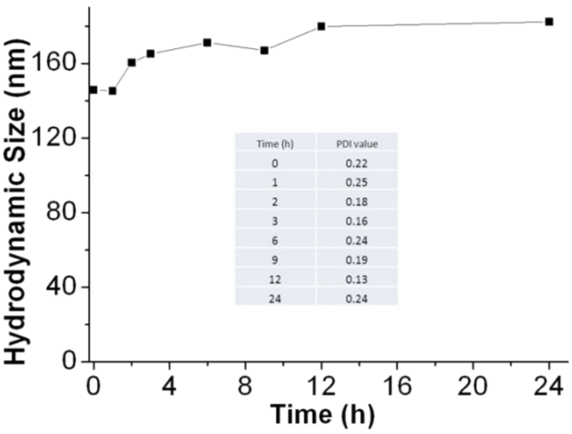

Fig. S7: Mean hydrodynamic diameter values of CPPNs in saline solution measured at 37 ^{o}C at different time intervals.

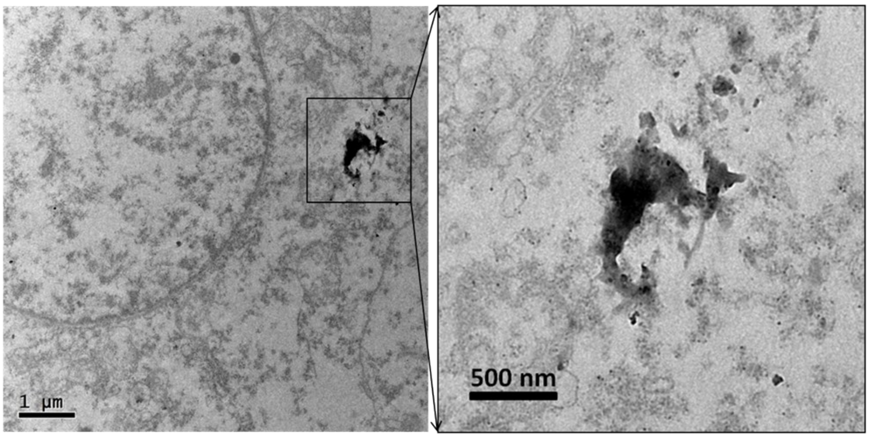

Fig. S8: TEM images of CPPNs internalized by 4T1 cells.

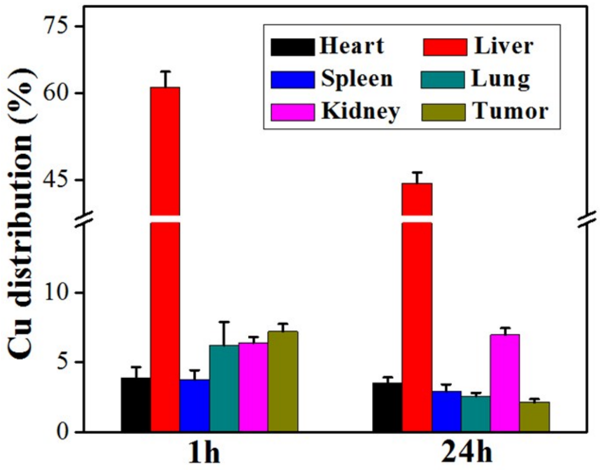

Fig. S9: The bio-distribution of Cu in 1 h and 24 h after intravenous injection of CPPNs.

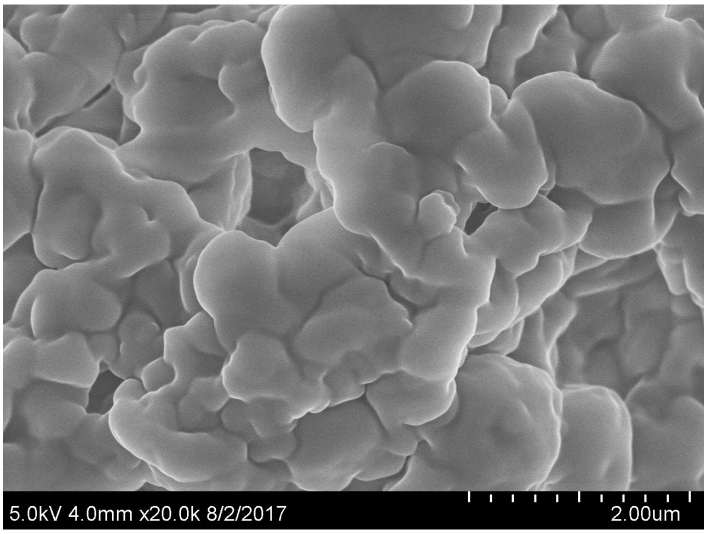

Fig. S10: SEM image of CPPNs after HIFU irradiation at 140 W for 10 s (5 s twice with a 5 s interval). The nanometer-sized spherical morphology of CPPNs mostly disappeared, and large micrometer dimension aggregations formed.

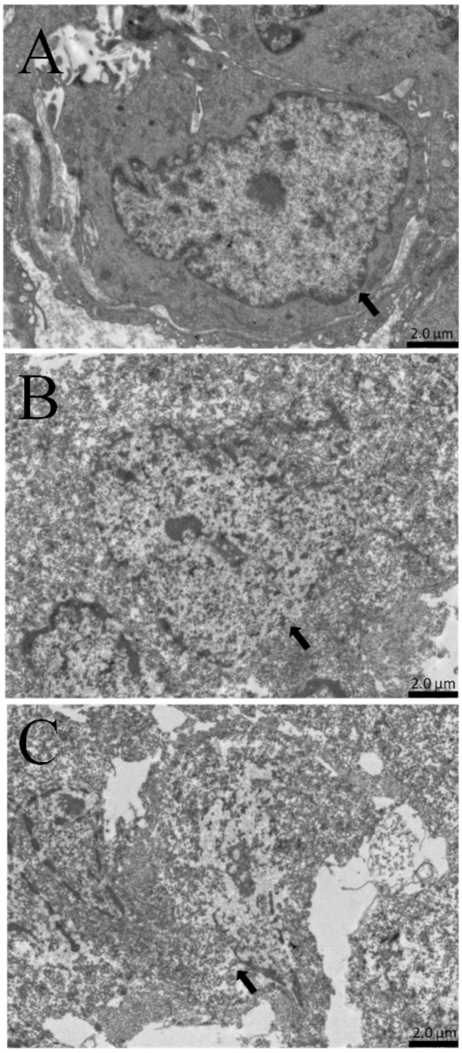

Fig. S11: TEM images of tumors in different groups after HIFU exposure at an acoustic power output of 140 W for 10 s (5 s twice, 5 s interval): (A) intravenous injection with saline; (B) intravenous injection of a 2.5 mg/mL CPPN suspension; (C) intravenous injection of a 5.0 mg/mL CPPN suspension. Arrows indicate the representative regions of the nuclear membrane. Notably, the arrow in Fig. S11-C shows irreversible damage of nuclear membrane.

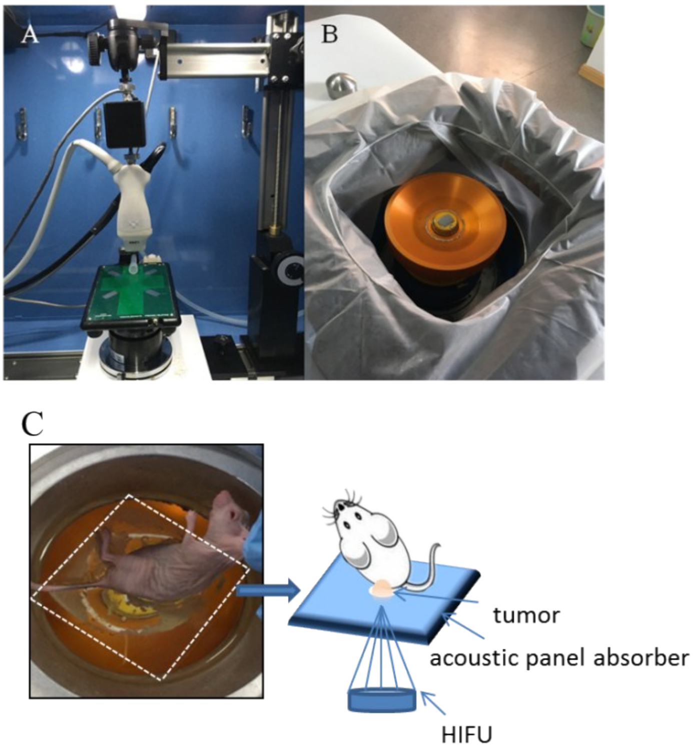

Fig. S12: (A, B) Photos of (A) PA imaging instrument and (B) HIFU treatment instrument. (C) Schematic diagram depicting the HIFU ablation process on a tumor-bearing mouse model. An acoustic panel absorber was employed to shield non-tumor tissues from acoustic exposure.
